# Supplementary material for: Uniaxial Strain Dependence on Angle-Resolved Optical Second Harmonic Generation from a Few Layers of Indium Selenide
Source: Nanomaterials (Basel). 2023 Feb 16;13(4):750. doi: 10.3390/nano13040750 (PMC9962579; doi:10.3390/nano13040750)
Supplement: Supplementary file 1 [file nanomaterials-13-00750-s001.zip › nanomaterials-2195152-supplementary.pdf]

Supplementary Material for
Uniaxial Strain Dependence on Optical Second Harmonic Generation from Indium Selenide Few-Layers

Zi-Yi Li[1,2], Hao-Yu Cheng[1,2], Sheng-Hsun Kung[1], Hsuan-Chun Yao[1], Christy Roshini Paul Inbaraj[2], Raman Sankar[1], Min-Nan Ou[1], Yang-Fang Chen[2], Chi-Cheng Lee[3*], Kung-Hsuan Lin[1*]

[1] Institute of Physics, Academia Sinica, Taipei 11529, Taiwan
[2] Department of Physics, National Taiwan University, Taipei 10617, Taiwan
[3] Department of Physics, Tamkang University, Tamsui, New Taipei 251301, Taiwan
* Email: cclee.physics@gmail.com (C.-C. Lee); linkh@sinica.edu.tw (K.-H. Lin)

(a) (b)

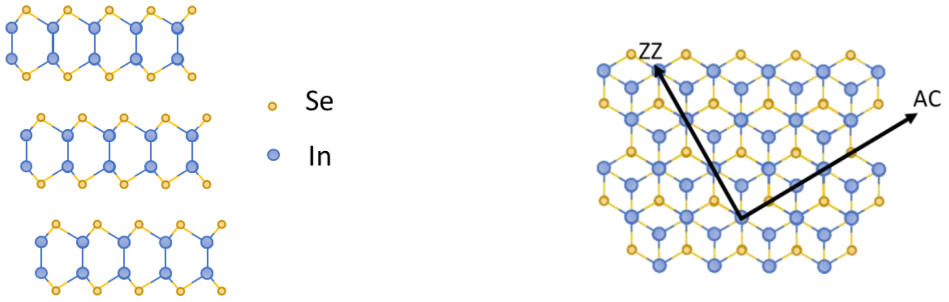

Figure S1. Structure of γ-phase InSe from the view along (a) [010] and (b) [001].

(a) (b)

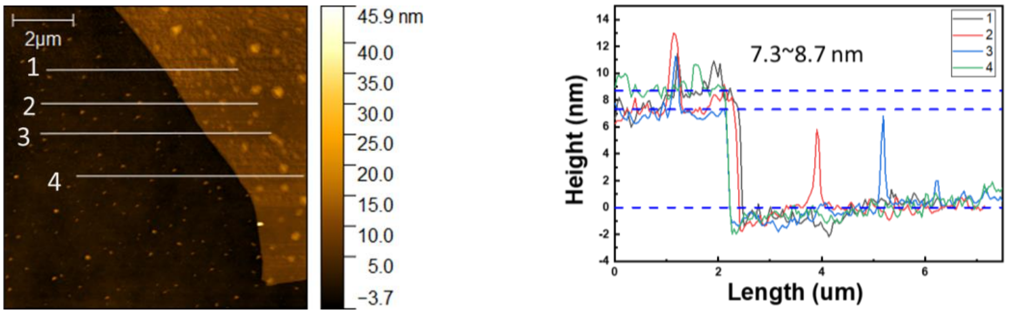

Figure S2. (a) AFM image around the edge of the InSe flake. (b) The 4 line profiles as labelled in (a).

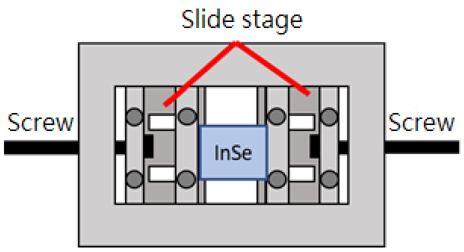

Figure. S3. Schematic of the strain device.

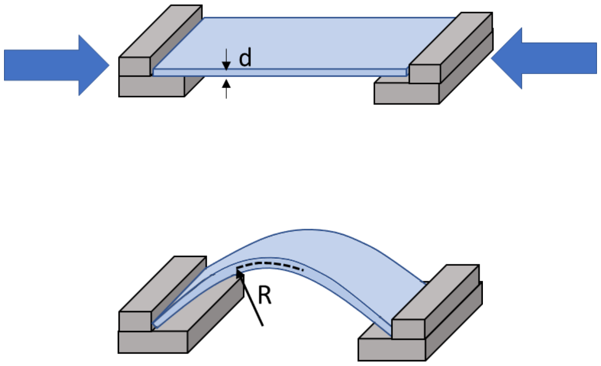

Figure S4. Schematic diagram of curved substrate under uniaxial strain.

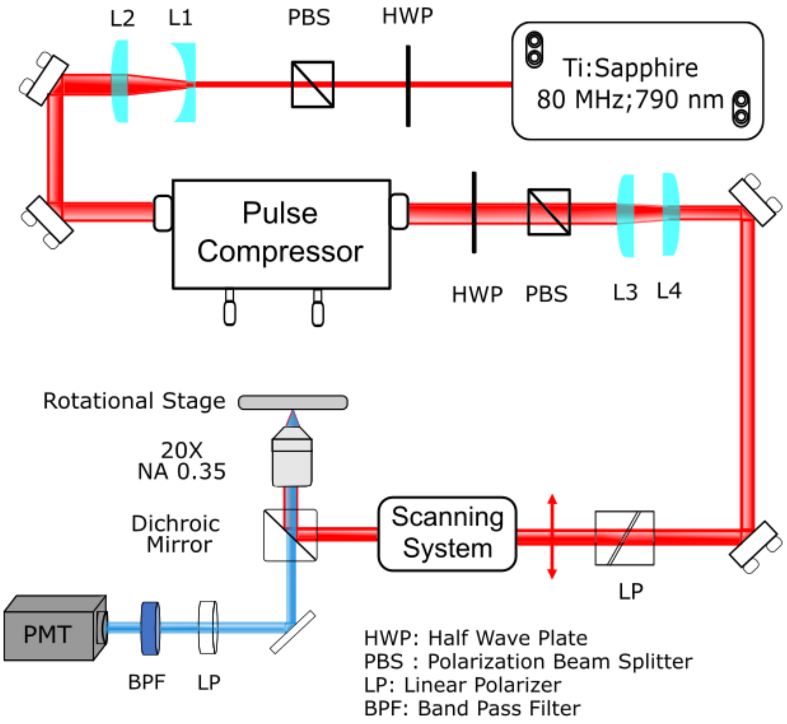

Figure S5. Schematic of the experimental setup.

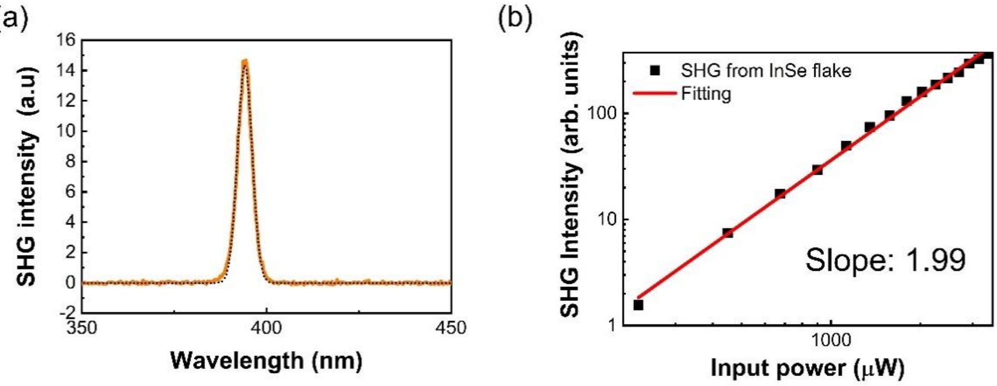

Figure S6. (a) The SHG spectrum with peak wavelength at 394 nm. (b) The power dependent SHG intensity. The slope of the linear fitting curve is around 2 in the log-log scale, indicating the quadratic relation between the incident power and the SHG intensity.

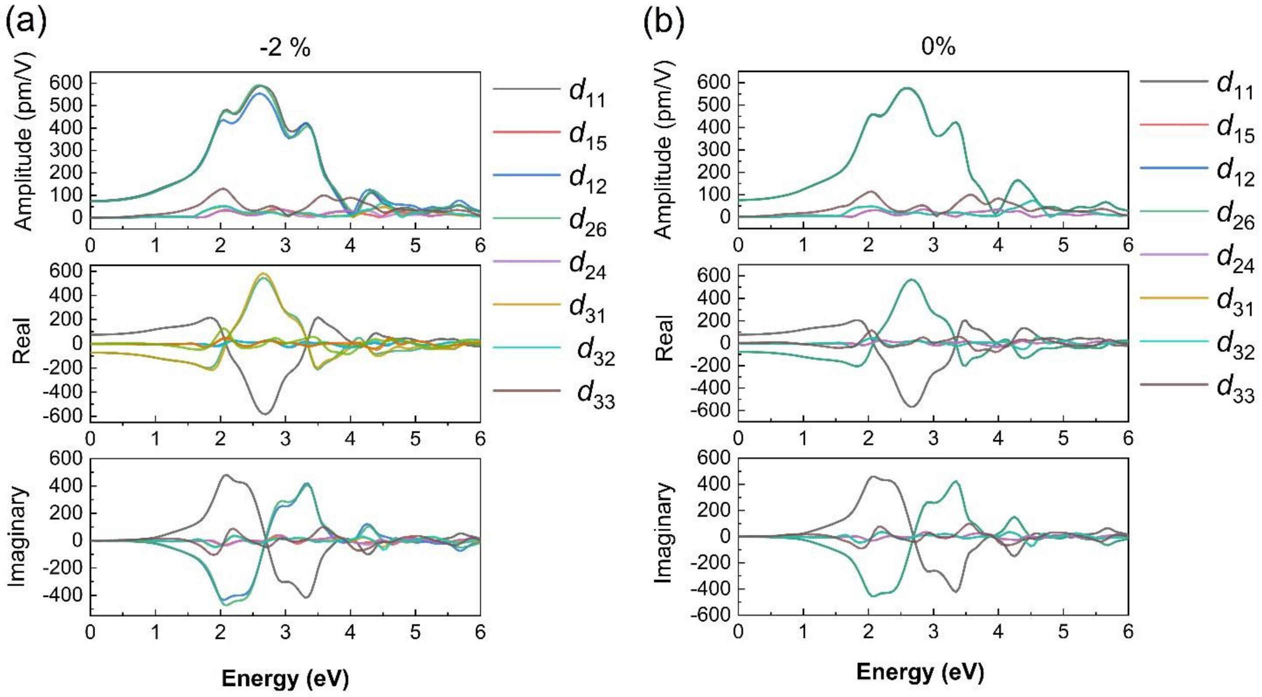

Figure S7. Calculated SHG susceptibility components of γ-InSe under strain of (a) -2% and (b) 0%.

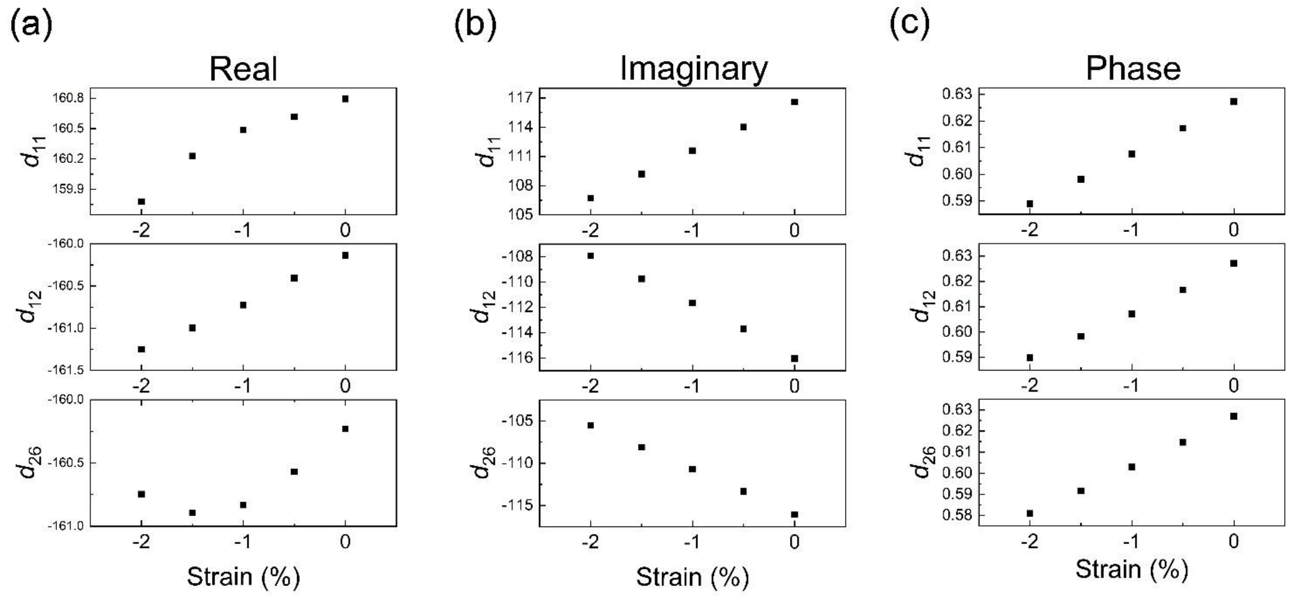

Figure S8 Strain dependence on the (a) real part, (b) imaginary part, and (c) phase of calculated d_{11}, d_{12} and d_{26} at 1.55 eV.

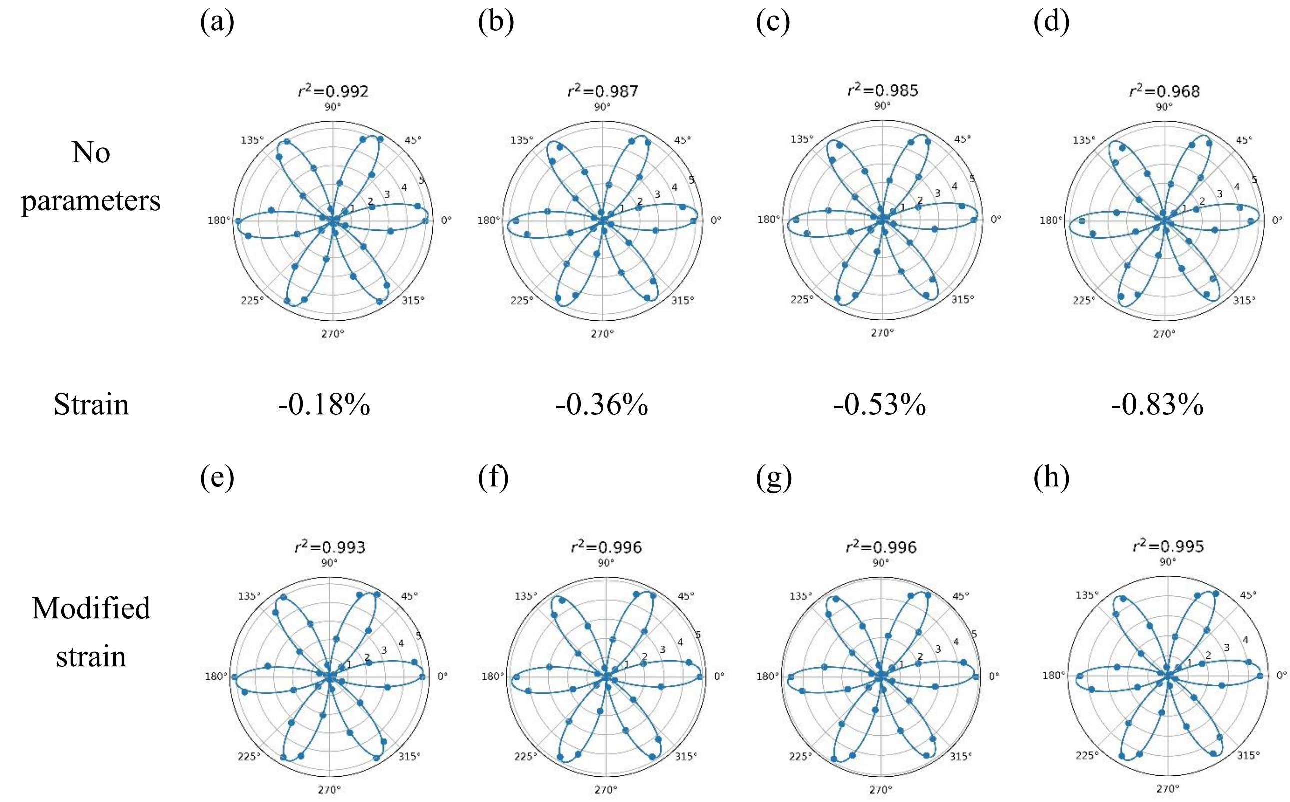

Figure S9. R-squared values of the strain-dependent experimental data by using the model from the first-principles methods (a)-(d) without parameters and (e)-(h) with one parameter to linearly modify the strain.
